# Determination of Total Tritium in Urine from Residents Living in the Vicinity of Nuclear Power Plants in Qinshan, China

**DOI:** 10.3390/ijerph120100888

**Published:** 2015-01-16

**Authors:** Bao-Ming Shen, Yan-Qin Ji, Qing Tian, Xiang-Zhang Shao, Liang-Liang Yin, Xu Su

**Affiliations:** China CDC Key Laboratory of Radiological Protection and Nuclear Emergency, National Institute for Radiological Protection, Chinese Center for Disease Control and Prevention, Beijing 100088, China; E-Mails: shenbm9@sina.com (B.-M.S.); bankvole@sohu.com (Q.T.); shaoxzh@nirp.cn (X.-Z.S.); yinliangliang2010@163.com (L.-L.Y.); suxu@nirp.cn (X.S.)

**Keywords:** urine, tritium, concentration of tritium, committed effective dose

## Abstract

To estimate the tritium doses of the residents living in the vicinity of a nuclear power plant, urine samples of 34 adults were collected from residents living near the Qinshan nuclear power plant. The tritium-in-urine (HTO plus OBT) was measured by liquid scintillation counting. The doses of tritium-in-urine from participants living at 2, 10 and 22 km were in a range of 1.26–6.73 Bq/L, 1.31–3.09 Bq/L and 2.21–3.81 Bq/L, respectively, while the average activity concentrations of participants from the three groups were 3.53 ± 1.62, 2.09 ± 0.62 and 2.97 ± 0.78 Bq/L, respectively. The personal committed effective doses for males were 2.5 ± 1.7 nSv and for females they were 2.9 ± 1.3 nSv. These results indicate that tritium concentrations in urine samples from residents living at 2 km from a nuclear power plant are significantly higher than those at 10 km. It may be the downwind direction that caused a higher dose in participants living at 22 km. All the measured doses of tritium-in-urine are in a background level range.

## 1. Introduction

Release of tritium into the environment from heavy water reactor operation is increasing the concentrations of tritium in the atmospheric water vapor and tritium occurs in the form of tritiated water vapor (HTO) and TH [1,2] that can easily been inhaled and absorbed by breathing and skin exposure. In addition, the distribution of tritium in precipitation and river water are important sources for human exposure. In the human body, the HTO and TH can quickly form free water tritium (FWT) and organically bound tritium (OBT) which are distributed in the tissues and organs in the uniform manner [3]. Previous studies demonstrated that tritium causes ionizing radiation damage primarily on the radiosensitive leukocyte cells [4]. Some studies [5,6] have shown that the OBT contributes to 5%–10% internal radiation dose in occupationally exposed workers, while residents around NPPs is 26%–50%, and the origin of OBT in body is dietary intake. Thus, monitoring the concentration of HTO and OBT in urine is important for the dose assessment of exposure to tritium. Here, the concentrations of tritium-in-urine (HTO plus OBT) from 34 participants living in the vicinity of a nuclear power plant were measured and the annual effective doses of exposure to tritium were evaluated as well.

## 2. Experimental Section

### 2.1. Sample collection and Preparation

Thirty four (34) participants who were living within 30 km of the Qinshan nuclear power plant were selected. The ages of the 34 residents ranged from 18 to 49 years old and the participants include 23 males and 11 females. The urine samples were collected between 10 pm to 6 am with a 120 mL sample cup. The collected samples were labeled and stored in fridge and treated the next day.

Before detection, 10 gram of potassium persulfate (99%, analytical grade, Acros Organics, NJ, USA) was added in a distillation apparatus to 50 mL of the collected urine samples. This mixture was oxidized for 30 min and a total of 20 mL of distillate was collected as described before [7]. Four mL of distillate and 14 mL of scintillation cocktail (Ultima Gold LLT, Perkin Elmer Inc., Waltham, MA, USA) were placed in a glass vial, and the same conditions were used in duplicate. To one vial 0.1 mL 1.5 Bq/mL tritium standard solution (China Institute of Atomic Energy, Beijing, China, specific activity 150 Bq /mL, uncertainty is 2.4%), was subsequently added , the mixture was shaken and stored in the dark for 72 h. Then the mixtures were determined by a TRI-CARB 3170 TR/SL (Perkin Elmer) liquid scintillation counter in lower level mode with the energy range 0~18.6 keV.

### 2.2. Sample Analysis

The concentration of tritium-in-urine (HTO plus OBT) was obtained with the following equation [7]:

(1) $$A=\left(N_{s}-N_{b}\right)/V\varepsilon$$

where *A*: the concentration of tritium-in-urine (HTO plus OBT), Bq/L; *V*: the volume of the urine sample using the measurement, L; *ε:* the counting efficiency of tritium, *ε* *= (N_a_ − N_s_)/D*, *N_a_*, tritium spiked sample count rate, counting·s^−1^; D, tritium activity spiked, Bq; *Ns*: count rate of the sample, counting·s^−1^; *N_b_*: the background count rate of the sample, counting·s^−1^. The limited detection concentration (*LDC*) and minimum detectable concentration (*MDC*) of tritium in urine [8] were defined as:

When the confidence level is 95%, α = β = 0.05, *K* = 1.65; *t_b_* = *t_s_*.

(2) $$LDC=2.33\sqrt{n_{b}/t_{b}}/V\varepsilon$$

and *MDC* = *2LDC*. Where the parameter *n_b_* = 0.035 s^−1^, *t_b_* = 60,000 s, *ε* = 35.2%, *V* = 4 × 10^−3^ L, the *LDC*= 1.26 Bq/L and *MDC* = 2.52 Bq/L, respectively.

The coefficient of variation (*CV*) for net counting and tritium counting efficiency are the main source of error in measuring the concentration of tritium-in-urine. The *CV* of tritium concentration in urine is in the range of 13% to 29% as the tritium concentration in urine is greater than the *MDC*. 

### 2.3. Committed Effective Dose

On the basis of adult intake of tritiated water, committed effective dose is estimated using the following parameters: total body mass is 73 kg for males and 60 kg for females. Water content of the body mass is 73% [9,10]. Dose coefficient is 1.8 × 10^−11^ Sv/Bq.

## 3. Results and Discussion

### 3.1. Concentration of Tritium in-Urine

The concentrations of tritium-in-urine from the 34 participants living in the vicinity of the Qinshan nuclear power plant were measured and the results were shown in Table 1. The concentration of tritium-in-urine was ranged from *LDC* to 6.73 Bq/L, its *CV* was 13–29% for greater than the detection limit (*MDC* = 2.52 Bq/L).

**Table 1:** The concentration of tritium-in-urine.

ID No.	Gender	(HTO plus OBT) (Bq/L)	*CV(%)*	ID No.	Gender	(HTO plus OBT) (Bq/L)	*CV(%)*
01	Male	6.16	14	18	Male	1.31	60
02	Male	3.75	23	19	Male	ND ^#^	
03	Female	3.91	20	20	Female	2.17	36
04	Female	3.23	25	21	Male	ND	
05	Female	6.73	13	22	Female	1.61	49
06	Male	3.82	20	23	Male	1.65	45
07	Male	ND		24	Female	ND	
08	Male	1.26	62	25	Male	2.27	36
09	Male	2.69	29	26	Female	2.81	30
10	Female	4.66	20	27	Male	1.79	45
11	Male	2.89	28	28	Male	ND	
12	Male	1.44	54	29	Male	3.81	23
13	Male	1.76	42	30	Female	ND	
14	Male	2.41	34	31	Female	2.41	35
15	Male	3.12	26	32	Male	3.46	25
16	Male	5.18	16	33	Female	2.21	35
17	Male	3.09	25	34	Male	ND	

Notes: ^#^ ND, means result is smaller than *LDC*.

**Figure 1:**
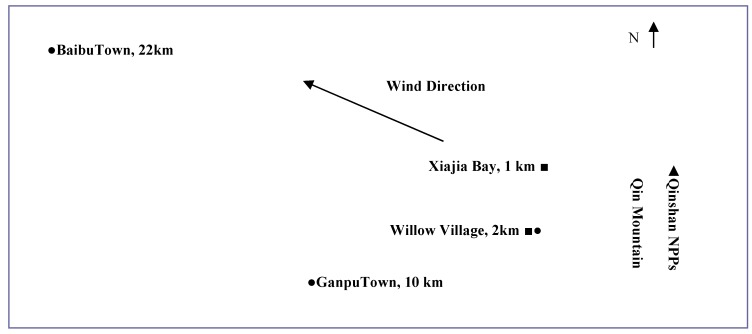
Sketch of wind direction, sampling points and monitoring sites. ■ Monitoring sites in reference 2; ● Sampling Point in this work.

The sampling points of residents living at 2, 10 and 22 km are shown in Table 2. The concentrations of tritium-in-urine (samples 1 to 16) from participants living at 2 km were in a range of 1.26–6.73 Bq/L, with an average value of 3.53 ± 1.62 Bq/L. The measurement of urine samples from 10 km (numbers 17 to 28) were 1.31–3.09 Bq/L, with an average value of 2.09 ± 0.62 Bq/L, while the concentration of tritium-in-urine from residents living at 22 km (numbers 29 to 34) ranged from 2.21–3.81 Bq/L and the average value was 2.97 ± 0.78 Bq/L. The results of urine tritium concentration in local residents were significantly different (*p* < 0.05, SPSS13.0, t = 3.065) between the 2 km and 10 km sampling points. As for the 22 km distance sampling point, the values showed no significant difference compared with the 2 km sampling point. The main reason was the dominant wind direction on the Zhejiang coast, which was a wind from March to August (Figure 1). The samples were collected in July, so the southeast wind affected tritium concentration of water and atmosphere in the region. Additionally, the Qin Mountain located at the southwest direction of Qinshan NPPs, which might be a natural barrier preventing tritium dispersion in southwest direction [2].

**Table 2:** The average concentration of tritium that populations living at different distances and orientation of urine samples.

ID No	N ^#^	Location	Range, HT and BT (Bq/L)	Mean, HT and BT (Bq/L)
01–16	15	Southwest, 2 km	1.26–6.73	3.53 ± 1.62
17–28	8	Southwest, 10 km	1.31–3.09	2.09 ± 0.62
29–34	4	Northwest, 22 km	2.21–3.81	2.97 ± 0.78

Notes: ^#^ The number of samples whose value were higher than LDC.

### 3.2. Dose of Tritium Contribution

The committed effective dose of participants was calculated on the basis of the measured results and the parameters for tritium recommended by the ICRP [9,10]. It was 2.5 ± 1.7 nSv for males and 2.9 ± 1.3 nSv for females.

**Table 3:** Tritium concentration in urine and committed effective doses for the exposed workers and the general public for tritium according to reported in the literature.

Country/Region	n	Mean (Bq/L)	Rang ^##^(Bq/L)	Committed Effective Dose *E* (nSv)		References
				Males	Females	
China/Sichuan			4.4–12.6			[11]
Canada/AECL^#^ southeast 0.6–200 km	5		6.5–53			[5]
Korea/who do not work at a nuclear facility	50	2.8 ± 1.4	1.8–5.6	1.9 ± 0.7	2.4 ± 1.1	[12]
Finland, Helsinki	227	2.55	1.5–18.3	2.4		[13]
China/Vicinity of NPPs	27	3.02 ± 1.42	1.26–6.73	2.5 ± 1.7	2.9 ± 1.3	This work

Notes: ^#^AECL-Chalk River Laboratories; ^##^ reported in the literature HTO, this work is HTO plus BTO.

## 4. Conclusions

With the increase of nuclear power plants in recent years, the release of tritium in the form of TH and tritiated water vapor from reactor operations into the environment is increasingly influencing the surrounding environment [2,5]. The results of this study showed that concentrations of total tritium-in-urines from participants living 2 km in the southwest direction from a nuclear power plant were significantly higher (*p* < 0.05, SPSS13.0, t = 3.065) than the average of urine samples from residents living at 10 km from the plant. The average concentration of tritium-in-urine from people living at 22 km from the nuclear power plant was 2.97 ± 0.78 Bq/L. This may due to the northwest direction which is the dominant downwind direction of the nuclear power plant, in which the orientation of atmospheric HTO and TH were significantly higher than in the southwest direction. The annual average tritium concentration of atmospheric of Xiajia Bay and Willow village (Figure 1) were 348 and 101 mBq/m^3^ [2].

Compared with the concentrations of tritium-in-urines from residents living in the vicinity of a decommissioning of nuclear facilities is in a range of 4.38–12.57 Bq/L [11], and from 50 Koreans who do not work at a nuclear facility it ranged 1.8–5.6 Bq/L [12], and the mean activity concentration of tritium in the urine of Finnish people is 2.55 Bq/L and the maximum 18.3 Bq/L [13]. Our results (1.26~6.73 Bq/L) show the similar ranges of tritium-in-urine (Table 3). The analytical method used measured total tritium and it does not therefore detect OBT. With tritiated water dose coefficients (1.8 × 10^−11^ Sv/Bq) [9] applied directly to the body effective dose calculated in this way may be underestimated. For males and females they were 2.5 ± 1.7 nSv and 2.9 ± 1.3 nSv, respectively. The reason is that the OBT dose coefficient (4.2 × 10^−11^ Sv/Bq) [9] is higher than for tritiated water. As such, the continuous chronic intake estimates of the dose due to the public are reasonable. In short, since these results were much lower than the dose limit for the public, background information is provided for reference only.
